# 937 Impact of Burn Injury Characteristics on Pediatric Acute Stress and Quality of Life

**DOI:** 10.1093/jbcr/iraf019.468

**Published:** 2025-04-01

**Authors:** Amanda Stevens, Nicole Caraballo, Elika Ridelman, Mariah Malaniak, Lisa Vitale, Justin Klein, Christina Shanti

**Affiliations:** Wayne State University School of Medicine; Children’s Hospital of Michigan Burn Center; Department of Surgery, Division of Pediatric Surgery, Children’s Hospital of Michigan, Wayne State University School of Medicine; Department of Surgery, Division of Pediatric Surgery, Children’s Hospital of Michigan, Wayne State University School of Medicine; Department of Surgery, Division of Pediatric Surgery, Children’s Hospital of Michigan, Wayne State University School of Medicine; Department of Surgery, Division of Pediatric Surgery, Children’s Hospital of Michigan, Wayne State University School of Medicine; Department of Surgery, Division of Pediatric Surgery, Children’s Hospital of Michigan, Wayne State University School of Medicine

## Abstract

**Introduction:**

Assessing psychological stress in children following burn injuries is crucial for providing comprehensive care. The Children’s Dermatology Life Quality Index (CDLQI) and the Acute Stress Checklist for Children, 6-item (ACS- 6) are validated tools that can be administered to evaluate quality of life and acute stress in pediatric burn patients. This study aims to examine the relationship between burn injury characteristics, acute stress, and quality of life in children aged 8 years and older following burn injuries.

**Methods:**

A retrospective chart review was conducted for pediatric burn patients (N=311) treated at a verified burn center between August 2021 and March 2024. Basic demographic data were collected as well as burn injury characteristics, and CDLQI and ASC-6 scores. Independent samples t-tests, one-way analyses of variance, and chi-square tests for independence were used to analyze burn injury characteristics with CDQLI and ASC-6 scores via the SPSS version 19 software.

**Results:**

A statistically significant difference was found in CDLQI scores related to total body surface area (TBSA) and the number of burn locations (p < 0.05). Significant associations were also found between CDLQI scores and debridement performed in the operating room (p < 0.05), and mechanism of injury (p < 0.001). There was a statistically significant increased likelihood of the ASC-6 exceeding clinical cutoff for burns to the head/neck (p< 0.05), burns to the torso (p < 0.05), female sex (p < 0.05), and younger age (p < 0.05). Additionally, there was a statistically significant relationship between the CDLQI and ASC scores (p < 0.001). No significant differences were observed based on race, ethnicity, insurance status, burn visibility, burn depth, or length of hospital stay.

**Conclusions:**

While higher quality of life scores (CDLQI) were associated with an increased likelihood of clinically significant acute stress (ASC-6), burn injury variables influenced each measure differently when analyzed independently. TBSA and the number of burn locations had a significant impact on quality of life but not on acute stress. Younger age, female gender, and burns to the head/neck and torso were more predictive of elevated acute stress scores.

**Applicability of Research to Practice:**

These findings can inform screening recommendations for quality of life and acute stress in pediatric burn patients. By identifying quality of life and acute stress as distinct constructs, this study highlights the importance of targeted psychological interventions post-injury.

**Funding for the Study:**

N/A

